# Managing Metabolic Dysfunction–Associated Steatotic Liver Disease: Protocol for a Scoping Review of Patient Perceptions, Barriers, and Facilitators

**DOI:** 10.2196/81404

**Published:** 2026-03-24

**Authors:** Sikyeong Park, Yu Shin Park, Dahye Hong, Bada Kang

**Affiliations:** 1 Yonsei University College of Nursing Seoul Republic of Korea; 2 Mo-Im Kim Nursing Research Institute Yonsei University College of Nursing Seoul Republic of Korea; 3 College of Nursing and Brain Korea 21 FOUR Project Yonsei University Seoul Republic of Korea; 4 Yonsei Evidence Based Nursing Centre of Korea: A JBI Affiliated Group Yonsei University College of Nursing Seoul Republic of Korea

**Keywords:** barriers, facilitators, health behaviors modification, metabolic dysfunction–associated steatotic liver disease, nonalcoholic fatty liver disease, patient perception

## Abstract

**Background:**

Metabolic dysfunction–associated steatotic liver disease (MASLD), formerly known as nonalcoholic fatty liver disease (NAFLD), is a major global health concern, affecting over 30% of adults worldwide. Closely associated with metabolic comorbidities such as obesity, type 2 diabetes, and dyslipidemia, MASLD relies heavily on health behavior modification for effective management. However, sustaining healthy behaviors remains challenging, particularly due to the disease’s asymptomatic nature in its early stages and low perceived severity among patients. Thus, understanding patient perceptions and identifying barriers and facilitators are essential for developing effective, patient-centered interventions.

**Objective:**

This scoping review protocol aims to systematically map the existing literature on patients’ perceptions of MASLD and to identify barriers and facilitators that influence disease management and patient engagement.

**Methods:**

This scoping review will follow the Joanna Briggs Institute scoping review methodology and the PRISMA-ScR (Preferred Reporting Items for Systematic Reviews and Meta-Analyses extension for Scoping Reviews) guidelines. A systematic search will be conducted in PubMed, CINAHL, Cochrane Library, and PsycINFO by using MeSH terms and keywords such as “MASLD,” “NAFLD,” “perception,” “awareness,” “attitude,” “barriers,” and “facilitators.” Two independent reviewers will screen studies, extract data using a standardized charting form, and resolve discrepancies through discussion or consultation with a third reviewer. Eligible studies will include adults with MASLD/NAFLD and will focus on patient perceptions, health beliefs, behaviors, or barriers and facilitators to management. Data synthesis will be guided by the socioecological model to categorize findings across individual, interpersonal, organizational, community, and public policy levels.

**Results:**

The research protocol was finalized in January 2026. This study received no external funding. A preliminary literature search was conducted by January 30, 2026, and formal screening of titles and abstracts is scheduled to begin in February 2026. Data extraction and synthesis are expected to be completed by April 2026. The final results are anticipated to be submitted for publication in the summer of 2026.

**Conclusions:**

This scoping review will offer a comprehensive overview of patients’ perceptions of MASLD, as well as the barriers and facilitators that influence its management. The results will inform the development of patient-centered strategies aimed at improving care delivery and reducing disease impact.

**International Registered Report Identifier (IRRID):**

DERR1-10.2196/81404

## Introduction

Metabolic dysfunction–associated steatotic liver disease (MASLD) has emerged as a significant global public health concern [1]. Defined by hepatic fat accumulation in the absence of significant alcohol consumption, MASLD is closely associated with metabolic comorbidities such as obesity, type 2 diabetes mellitus, and dyslipidemia [2]. Recent estimates indicate that MASLD affects over 30% of the global adult population, underscoring its high prevalence and clinical significance [3]. While the early stages are typically asymptomatic, MASLD can progress to more severe conditions, including nonalcoholic steatohepatitis, advanced fibrosis, cirrhosis, and hepatocellular carcinoma, underscoring the critical need for early detection and timely intervention [4]. Recently, a multi-society Delphi consensus renamed nonalcoholic fatty liver disease (NAFLD) to MASLD. This change reflects the adoption of affirmative diagnostic criteria that better represent the underlying pathophysiology and aims to remove stigmatizing language associated with the terms “fatty” and “nonalcoholic” [5].

Globally, MASLD imposes a substantial and growing health care and economic impact. This trend is also evident in South Korea, where prevalence estimates range from 10% to 50%, depending on diagnostic criteria and study populations [6]. According to data from the National Health Insurance Service of the Republic of Korea, the incidence of MASLD increased from 1.87% in 2010 to 4.47% in 2022, with projections estimating a prevalence of 43.8% by 2035 [7]. This rising impact extends beyond epidemiology, posing significant health care and economic challenges. The National Health Insurance Service claims analyses reveal that annual medical expenditures for individuals with MASLD are approximately 44% higher than those without the condition (KRW 787,579 [US $531] vs KRW 548,494 [US $327], respectively), largely due to greater pharmaceutical use and health care service utilization [8]. Likewise, data from the Korean Health Insurance Review and Assessment Service demonstrate that the prevalence of MASLD nearly doubled from 15.7 to 34.2 per 1000 population between 2010 and 2021, while the prevalence of metabolic dysfunction–associated steatohepatitis increased nearly 20-fold during the same period [9].

Health behavior modification, including dietary changes, increased physical activity, and weight reduction, is widely recognized as the cornerstone of MASLD management [10-13]. Sustained adherence to these behaviors has been shown to improve hepatic steatosis, reverse early-stage fibrosis, and reduce the risk of progression to advanced liver disease [14,15]. Despite these benefits, maintaining long-term health behavior changes remains a major challenge. Studies consistently report suboptimal adherence rates to dietary and exercise guidelines [16-18].

A growing body of literature has explored factors influencing patient engagement in MASLD management, identifying both barriers and facilitators. However, no comprehensive synthesis has systematically mapped the psychosocial, cultural, and structural factors that influence patient engagement in MASLD care. Existing studies typically focus on specific interventions or isolated populations, with limited attention to the broader sociocultural context shaping patient behaviors [10,19-21]. Moreover, much of the available evidence originates from Western populations, leaving significant gaps in understanding how sociocultural factors, health care systems, and patient beliefs affect disease management in other settings, particularly in Asian contexts such as South Korea [8,22,23]. Addressing these knowledge gaps is essential for informing the development of culturally sensitive, patient-centered interventions and public health strategies aimed at improving long-term outcomes for individuals living with MASLD.

Given these considerations, this scoping review will systematically map the existing literature on (1) how individuals with MASLD perceive the disease, (2) barriers that hinder effective management, and (3) facilitators that support sustained engagement and adherence to health behavior interventions. By synthesizing this evidence, the review seeks to provide a foundational understanding of factors that can inform the development of culturally tailored interventions and guide comprehensive public health responses to the growing impact of MASLD.

## Methods

### Overview

This scoping review will be conducted in accordance with the Joanna Briggs Institute (JBI) methodological framework [24]. The PRISMA-ScR (Preferred Reporting Items for Systematic reviews and Meta-Analyses extension for Scoping Reviews) checklist will be used for reporting [25]. A structured approach will be used to systematically identify, evaluate, and map relevant literature on patient perceptions and barriers and facilitators influencing MASLD management.

### Eligibility Criteria

To determine the eligibility criteria of the studies to be included in this review, we will apply the *Population, Concept, Context* framework for scoping review as outlined by JBI. The inclusion and exclusion criteria are specified as follows.

#### Population

The review will include adults (aged ≥18 years) diagnosed with NAFLD or MASLD based on either clinical confirmation or self-reported diagnosis, as explicitly defined in the included studies.

#### Concept

This review will include studies that examine how individuals with MASLD perceive the disease, encompassing their awareness, knowledge, beliefs, and attitudes toward the disease. It will also consider studies that explore barriers and facilitators influencing patient engagement in disease management and adherence to health behavior changes. Specifically, by structuring identified determinants into perceptions, barriers and facilitators within the socioecological model (SEM) framework, this review aims to provide a comprehensive understanding of the psychological, behavioral, and sociocultural factors affecting self-management behaviors.

#### Context

There will be no restriction on geographic location or health care settings, as the aim is to provide a comprehensive understanding of the literature across diverse cultural and health care contexts.

#### Types of Studies

This scoping review will consider a wide range of study designs. Eligible studies will include observational designs such as case reports, case-control studies, and cohort studies, as well as experimental designs including quasi-experimental studies and randomized controlled trials. In addition, qualitative observational studies and mixed methods studies that integrate both observational and experimental components will be considered. Scientific conference proceedings that report sufficient methodological detail and outcome data to support extraction and synthesis will be eligible for inclusion. To ensure that the review reflects contemporary evidence from the past decade, only studies published in English from 2016 onward will be included. Editorials, letters, commentaries, and books will be excluded.

#### Exclusion Criteria

Studies will be excluded if they focus exclusively on pharmacological, genetic, or physiological aspects of MASLD without addressing patient perspectives or behavioral factors. Regarding the study population, studies will be excluded if they do not involve individuals explicitly diagnosed with MASLD or NAFLD, or if they include mixed populations (eg, combined samples of MASLD and other liver diseases) or participants with significant alcohol consumption, unless data specific to the MASLD/NAFLD subgroup can be clearly disaggregated. Furthermore, studies that fail to explicitly describe barriers and facilitators to disease management or patient engagement will also be excluded. Additionally, studies that examine only health care providers’ perspectives without incorporating patient-level insights will not be considered.

### Data Sources and Search Strategy

The search strategy for this scoping review will follow the JBI 3-step approach. First, a preliminary search of PubMed and CINAHL was conducted to identify relevant keywords and Medical Subject Headings (MeSH) terms related to the research question. Based on this initial search, a comprehensive electronic search will be performed across the following databases: PubMed (MEDLINE), CINAHL, Cochrane Library, and PsycINFO. The search will incorporate both MeSH terms and free-text keywords related to the population and core concepts of interest, including but not limited to “metabolic dysfunction–associated steatotic liver disease (MASLD)” OR “nonalcoholic fatty liver disease (NAFLD)” AND “perception,” “awareness,” “attitude,” “belief,” “barriers,” “limitations,” “facilitators,” “enabler,” “behavior,” and “self-management.”

Boolean operators (AND/OR), truncation, and database-specific indexing will be applied to optimize the search. The specific keywords and controlled vocabulary terms (eg, MeSH) used are listed in Multimedia Appendix 1. The complete search strategies for all databases, including PubMed, CINAHL, Cochrane Library, and PsycINFO, are detailed in Multimedia Appendix 2.

### Screening and Selection Procedures of Eligible Studies

All records identified through the search will be imported into EndNote 20 (Clarivate Analytics) for reference management, and duplicate entries will be removed. Two independent reviewers will screen the titles and abstracts of retrieved studies for relevance based on the inclusion and exclusion criteria. Full texts of potentially eligible articles will then be assessed independently. Any disagreements during the selection process will be resolved through discussion or, if necessary, consultation with a third reviewer. The study selection process will be documented using a PRISMA-ScR flow diagram.

### Data Extraction and Synthesis

Data extraction will use a standardized charting form aligned with JBI guidelines. Two reviewers will independently extract data, including study characteristics (author, year, country, design, objectives, setting, and population details) and key concepts (patient perceptions, barriers, facilitators). Outcomes, key findings, study limitations, and author recommendations will also be documented (Multimedia Appendix 3). A standardized Microsoft Excel spreadsheet will be used to organize the extracted data.

To ensure a structured synthesis, the analysis will be guided by SEM [26]. Patient perceptions, barriers, and facilitators across included studies will be systematically coded and categorized into 5 distinct SEM levels: individual, interpersonal, organizational, community, and public policy (Multimedia Appendix 4). This framework-based approach allows for a stratified analysis of the factors influencing MASLD management, moving beyond a purely narrative summary to identify recurring patterns and evidence across SEM levels.

### Ethical Considerations

As this is a review of previously published literature, ethical approval from the institutional review board is not required.

## Results

The research protocol was finalized in January 2026. This study received no external funding. A preliminary literature search was conducted by January 30, 2026, and formal screening of titles and abstracts is scheduled to begin in February 2026. Data extraction and synthesis are expected to be completed by April 2026. The final results are anticipated to be submitted for publication in the summer of 2026.

## Discussion

This scoping review is expected to provide a foundational understanding of patient perceptions and the barriers and facilitators influencing the implementation of management strategies for MASLD. Although health behavior interventions—such as exercise programs, dietary modifications [10], and digital health strategies [20]—have demonstrated efficacy in controlled research settings, their translation into clinical practice remains limited.

In the broader context of chronic disease management, a substantial body of evidence supports the role of facilitators such as culturally tailored educational interventions [27], digital health technologies [28], and integrated multidisciplinary care models in improving patient engagement, treatment adherence, and clinical outcomes [29]. For instance, in conditions like type 2 diabetes, cardiovascular disease, and obesity, culturally sensitive education has been shown to enhance health literacy, reduce stigma, and promote sustainable behavior change across diverse populations [30-32]. Similarly, digital health platforms—including mobile apps, telemonitoring, and remote coaching—have improved access to care, supported self-management, and facilitated real-time feedback, especially for patients with limited physical access to clinical services [28]. Multidisciplinary care models that incorporate physicians, nurses, dietitians, and behavioral health professionals have also proven effective in delivering coordinated, holistic care for complex chronic conditions [29].

These facilitators are particularly applicable to MASLD, where long-term management heavily depends on patient-driven health behavior changes and sustained engagement with nonpharmacologic interventions [11]. Culturally adapted educational content may enhance disease awareness, encourage health behavior changes, and reduce barriers related to health beliefs or stigma. Digital tools can offer scalable, personalized support for behavior tracking, goal setting, and continuous monitoring outside of clinical visits [20]. Integrated care teams can provide comprehensive and coordinated support that addresses not only hepatic health, but also the psychological, nutritional, and metabolic dimensions of MASLD [33]. Together, these approaches highlight the importance of aligning intervention strategies with the diverse needs, preferences, and contexts of patients to improve the real-world implementation and effectiveness of MASLD management.

Previous studies have identified implementation challenges, including time and resource constraints, limited provider training, and insufficient infrastructure to support sustained patient engagement [34,35]. Although digital health interventions offer promising support for MASLD management, their scalability and adoption are often hindered by system-level barriers such as technical challenges and regulatory constraints. These limitations are further exacerbated by patient-level factors, including limited disease awareness, low intrinsic motivation for behavioral change, and sociocultural or systemic influences, all of which hinder engagement and adherence, thereby diminishing the overall effectiveness of such interventions [33].

These anticipated findings highlight the need for a deeper understanding of multilevel implementation factors influencing MASLD management. This scoping review aims to comprehensively identify and map the psychosocial, cultural, and structural determinants that influence MASLD management. The findings are expected to inform the development of tailored strategies to enhance the uptake, patient engagement, adherence, and long-term effectiveness of interventions across diverse health care settings. By mapping the interplay among patient perceptions, contextual barriers, and facilitators, the study seeks to inform the design of patient-centered approaches that are both feasible and sustainable in real-world practice.

Furthermore, this integrated understanding has implications beyond individual-level behavior change, extending to health system strengthening and policy development. In light of the increasing prevalence of MASLD in South Korea and globally [3,6], the findings from this review may provide empirical evidence to guide national strategies focused on early detection and prevention, the development of provider-facing education and counseling guidelines, and the implementation of public health campaigns aimed at improving health literacy. Collectively, these outcomes will contribute to a multilevel framework for advancing MASLD care and improving long-term patient outcomes.

However, several limitations should be considered. First, the heterogeneity of the included studies across diverse health care systems and sociocultural settings may limit the generalizability of these findings. Second, as a scoping review, this study focuses on mapping existing evidence rather than critically appraising study quality or establishing causality, which limits its direct applicability to clinical guideline development. Finally, this protocol was limited by the absence of patient involvement in the design or search strategy development phases. While this review aims to synthesize patient perspectives reported in the existing literature, the lack of direct consultation with individuals living with MASLD may limit the immediate applicability of the findings. Importantly, such research and subsequent intervention development should prioritize the active engagement of patients to ensure clinical and contextual relevance.

Future research should prioritize context-specific studies across different regions to explore patients’ perceptions and cultural influences on disease management. Moreover, identifying and evaluating evidence-based interventions tailored to local sociocultural environments will play a critical role in addressing the global impact of MASLD and improving long-term patient outcomes.

In conclusion, this scoping review protocol outlines a systematic and theory-informed approach to identifying the psychosocial, cultural, and structural determinants that influence MASLD management. By mapping the interplay among patient perceptions, contextual barriers, and facilitators using SEM, the review aims to inform the development of patient-centered strategies that are both feasible and sustainable in real-world clinical practice. Ultimately, the findings are expected to contribute to a comprehensive, multilevel framework for advancing MASLD care and to help bridge the gap between clinical recommendations and the lived experiences of patients.

## Appendix

Multimedia Appendix 1 Specific keywords and controlled vocabulary terms used for this study.

Multimedia Appendix 2 Search strategies.

Multimedia Appendix 3 Data extraction instrument.

Multimedia Appendix 4 Data synthesis instrument.

## Abbreviations

JBI: Joanna Briggs Institute
MASLD: metabolic dysfunction–associated steatotic liver disease
MeSH: Medical Subject Headings
NAFLD: nonalcoholic fatty liver disease
PRISMA-ScR: Preferred Reporting Items for Systematic reviews and Meta-Analyses extension for Scoping Reviews
SEM: socioecological model
