# Supplementary material for: Managing Metabolic Dysfunction–Associated Steatotic Liver Disease: Protocol for a Scoping Review of Patient Perceptions, Barriers, and Facilitators
Source: JMIR Res Protoc. 2026 Mar 24;15:e81404. doi: 10.2196/81404 (PMC13058532; doi:10.2196/81404)
Supplement: Multimedia Appendix 1 [file resprot_v15i1e81404_app1.docx]

**Multimedia Appendix 1**

**Supplementary Table 1. Keywords**

|  | Population | Concept | | |
| --- | --- | --- | --- | --- |
| Keywords | "MASLD"  "NAFLD"  "Non-alcoholic fatty liver disease"  "Nonalcoholic fatty liver disease"  "Metabolic dysfunction-associated steatotic liver disease"  “Patient” | perception  awareness  understanding  recognition  knowledge  belief  attitude  health literacy  consciousness | barrier*  challenge*  difficult*  hindrance*  limitation* | facilitator*  enabler*  supporting factor*  motivator*  promoter*  positive factor*  enabling condition* |
| Mesh | Non-alchoholic fatty liver disease[MeSH] |  |  |  |
| Database | PubMed, CINAHL, Cochrane library, PsycINFO, Embase | | | |
