# Supplementary material for: Managing Metabolic Dysfunction–Associated Steatotic Liver Disease: Protocol for a Scoping Review of Patient Perceptions, Barriers, and Facilitators
Source: JMIR Res Protoc. 2026 Mar 24;15:e81404. doi: 10.2196/81404 (PMC13058532; doi:10.2196/81404)
Supplement: Multimedia Appendix 2 [file resprot_v15i1e81404_app2.docx]

**Multimedia Appendix 2**

**Supplementary Table 1.** PubMed Search Strategy

| Search # | MeSH Terms and Keywords |
| --- | --- |
| #1 | "non alcoholic fatty liver disease"[MeSH Terms] |
| #2 | "MASLD"[Title/Abstract] OR "NAFLD"[Title/Abstract] OR "Non-alcoholic fatty liver disease"[Title/Abstract] OR "Metabolic dysfunction-associated steatotic liver disease"[Title/Abstract] OR "Nonalcoholic fatty liver disease"[Title/Abstract] |
| #3 | "perception"[Title/Abstract] OR "awareness"[Title/Abstract] OR "understanding"[Title/Abstract] OR "recognition"[Title/Abstract] OR "knowledge"[Title/Abstract] OR "belief"[Title/Abstract] OR "attitude"[Title/Abstract] OR "health literacy"[MeSH Terms] OR "health literacy"[Title/Abstract] OR "consciousness"[Title/Abstract] |
| #4 | "barrier*"[Title/Abstract] OR "challenge*"[Title/Abstract] OR "difficult*"[Title/Abstract] OR "hindrance*"[Title/Abstract] OR "limitation*"[Title/Abstract] |
| #5 | "facilitator*"[Title/Abstract] OR "enabler*"[Title/Abstract] OR "supporting factor*"[Title/Abstract] OR "motivator*"[Title/Abstract] OR "promoter*"[Title/Abstract] OR "positive factor*"[Title/Abstract] OR "enabling condition*"[Title/Abstract] |
| #6 | "patient*"[Title/Abstract] |
| #7 | (#1 OR #2) AND (#3 OR #4 OR #5) AND #6 |
| #8 | #7 Filters: from 2016 - |

**Supplementary Table 2.** CINAHL Search Strategy

| Search # | Subject Headings (MH) and Keywords |
| --- | --- |
| #1 | MH "Nonalcoholic Fatty Liver Disease" |
| #2 | TI ("MASLD" OR "NAFLD" OR "Non-alcoholic fatty liver disease" OR "Nonalcoholic fatty liver disease" OR "Metabolic dysfunction-associated steatotic liver disease") OR XB ("MASLD" OR "NAFLD" OR "Non-alcoholic fatty liver disease" OR "Nonalcoholic fatty liver disease" OR "Metabolic dysfunction-associated steatotic liver disease") |
| #3 | MH "Perception" or MH "Self-Awareness" or MH "Knowledge" or MH "Health Beliefs" or MH "Attitude" or MH "Health Literacy" or MH "Consciousness" |
| #4 | TI ("perception" OR "awareness" OR "understanding" OR "recognition" OR "knowledge" OR "belief" OR "attitude" OR "health literacy" OR "consciousness") OR XB ("perception" OR "awareness" OR "understanding" OR "recognition" OR "knowledge" OR "belief" OR "attitude" OR "health literacy" OR "consciousness") |
| #5 | TI ("barrier*" OR "challenge*" OR "difficult*" OR "hindrance*" OR "limitation*") OR XB ("barrier*" OR "challenge*" OR "difficult*" OR "hindrance*" OR "limitation*") |
| #6 | (MH "Motivation" or MM "Promoters") AND MM "Promoters" |
| #7 | TI ("facilitator*" OR "enabler*" OR "supporting factor*" OR "motivator*" OR "promoter*" OR "positive factor*"OR "enabling condition*") OR XB ("facilitator*" OR "enabler*" OR "supporting factor*" OR "motivator*" OR "promoter*" OR "positive factor*"OR "enabling condition*") |
| #8 | TI "patient*" OR XB "patient*" |
| #9 | #1 or #2 |
| #10 | #3 or #4 |
| #11 | #6 or #7 |
| #12 | #9 and #10 and #11 and #5 and #8 |
| #13 | #12 Filters: from 2016 - |

**Supplementary Table 3.** Cochrane Library Search Strategy

| Search # | MeSH Terms and Keywords |
| --- | --- |
| #1 | MeSH descriptor: [Non-alcoholic Fatty Liver Disease] explode all trees |
| #2 | MASLD OR "NAFLD" OR "Non-alcoholic fatty liver disease" OR "Nonalcoholic fatty liver disease" OR "Metabolic dysfunction-associated steatotic liver disease" |
| #3 | MeSH descriptor: [Perception] explode all trees |
| #4 | MeSH descriptor: [Knowledge] explode all trees |
| #5 | MeSH descriptor: [Comprehension] explode all trees |
| #6 | MeSH descriptor: [Attitude] explode all trees |
| #7 | MeSH descriptor: [Health Literacy] explode all trees |
| #8 | MeSH descriptor: [Consciousness] explode all trees |
| #9 | Perception or "Self-Awareness" or "Knowledge" or "Health Beliefs" or "Attitude" or "Health Literacy" or "Consciousness" |
| #10 | #3 or #4 or #5 or #6 or #7 or #8 or #9 |
| #11 | barrier OR "barriers" OR "challenge" OR "challenges" OR "difficulty" OR "difficulties" OR "hindrance" OR "hindrances" OR "limitation" OR "limitations" |
| #12 | MeSH descriptor: [Motivation] explode all trees |
| #13 | “facilitator” OR "facilitators" OR "enabler" OR "enablers" OR "supporting factor" OR "supporting factors" OR "motivator" OR "motivators" OR "promoter" OR "promoters" OR "positive factor" OR "positive factors" OR "enabling condition" |
| #14 | MeSH descriptor: [Patients] explode all trees |
| #15 | patient OR "patients" |
| #16 | #1 or #2 |
| #17 | #12 or #13 |
| #18 | #14 or #15 |
| #19 | #10 or #11 or #17 |
| #20 | #16 and #18 and #19 |
| #21 | #20 Filters: from 2016 - |

**Supplementary Table 4.** PsycINFO Search Strategy

| Search # | MeSH Terms and Keywords |
| --- | --- |
| #1 | "tiab(MASLD OR NAFLD OR Non-alcoholic fatty liver disease OR Nonalcoholic fatty liver disease OR Metabolic dysfunction-associated steatotic liver disease)" |
| #2 | tiab(perception OR awareness OR understanding OR recognition OR knowledge OR belief OR attitude OR health literacy OR consciousness) |
| #3 | tiab("barrier*" OR "challenge*" OR "difficult*" OR "hindrance*" OR "limitation*") |
| #4 | tiab("facilitator*"OR "enabler*" OR "supporting factor*" OR "motivator*" OR "promoter*" OR "positive factor*" OR "enabling condition*") |
| #5 | tiab("patient*") |
| #6 | #1 AND (#2 OR #3 OR #4) AND #5 |
| #7 | #6 Filters: from 2016 - |
