# Supplementary material for: Managing Metabolic Dysfunction–Associated Steatotic Liver Disease: Protocol for a Scoping Review of Patient Perceptions, Barriers, and Facilitators
Source: JMIR Res Protoc. 2026 Mar 24;15:e81404. doi: 10.2196/81404 (PMC13058532; doi:10.2196/81404)
Supplement: Multimedia Appendix 3 [file resprot_v15i1e81404_app3.docx]

**Multimedia Appendix 3**

Data extraction instrument

| **Author** | **Year** | **Title** | **Type of publication** | **Study design** | **Methods** | **Aim** | **Setting/**  **Country** | **Study participants** | **Additional findings /Comments** |
| --- | --- | --- | --- | --- | --- | --- | --- | --- | --- |
|  |  |  |  |  |  |  |  |  |  |
|  |  |  |  |  |  |  |  |  |  |
|  |  |  |  |  |  |  |  |  |  |
|  |  |  |  |  |  |  |  |  |  |
|  |  |  |  |  |  |  |  |  |  |
|  |  |  |  |  |  |  |  |  |  |
|  |  |  |  |  |  |  |  |  |  |
|  |  |  |  |  |  |  |  |  |  |
|  |  |  |  |  |  |  |  |  |  |
