# Supplementary material for: Managing Metabolic Dysfunction–Associated Steatotic Liver Disease: Protocol for a Scoping Review of Patient Perceptions, Barriers, and Facilitators
Source: JMIR Res Protoc. 2026 Mar 24;15:e81404. doi: 10.2196/81404 (PMC13058532; doi:10.2196/81404)
Supplement: Multimedia Appendix 4 [file resprot_v15i1e81404_app4.docx]

| **SEM Level** | **Perceptions** | **Barriers** | **Facilitator** |
| --- | --- | --- | --- |
| **Individual** |  |  |  |
| **Interpersonal** |  |  |  |
| **Organization/Health system** |  |  |  |
| **Community** |  |  |  |
| **Public policy** |  |  |  |

**Multimedia Appendix 4**

Data synthesis instrument
